# Regulated the electrokinetic application of different plant growth stages and parameters enhance the economic extraction of soil heavy metals

**DOI:** 10.3389/fpls.2025.1557261

**Published:** 2025-05-19

**Authors:** Hongyan Ma, Ming Zhao, Na Yang, Linhui Feng, Lirong Wang, Chen Jiang, Ming Jiang, Jianfang Guo, Tianguo Li

**Affiliations:** College of Resources and Environment, Yunnan Agricultural University, Kunming, China

**Keywords:** farmland contaminated soil, electrokinetic-assisted phytoremediation, strategy optimization, economic extraction, heavy metal

## Abstract

**Introduction:**

Electrokinetic-assisted phytoremediation (EKAPR) improved the heavy metal accumulation has been extensively covered, but the uneconomic of heavy metal extraction increment unit energy consumption (EHME) limits its development.

**Methods:**

The feasibility from the dual perspectives of regulated the electrokinetics application of different growth stages of Sedum plumbizincicola and electrochemical parameters affecting power consumption to enhance the EHME of EKAPR system were investigated.

**Results:**

Results shown that electrokinetic promoted heavy metals accumulation of S. plumbizincicola significantly, and it not show positive correlation absolutely with the application time. EK-B treatment exhibited high performance for Cu and Pb. Although the Cd and Zn extraction decreased 9.02%-15.63% for EK-B and EK-S compare with EK-W treatment due to difference in biomass, there was insignificant in the content. Comprehensive consideration of growth and accumulate characteristics, electrokinetic application in the booming stage (0.70 of PCA score) was alternative to replace whole growth period treatment. Orthogonal experiments results showed that four factors were insignificant with biomass, heavy metal content and extraction, while voltage gradients and application time had significant effect. The biomass and heavy metal extraction showed appropriate promoted effect in 1.5-2.5 V/cm, 100–150 h, whereas EHME continuous deceased and the decline rate relatively slow within 1.0V/cm, 100h. The result indicates the existence of optimization strategy, the best recommended strategy was T7 treatment, followed by T3, T8 and T12 treatments.

**Discussion:**

Overall, it is an acceptable option to study energy saving in terms of optimization of plant growth stage and electric field parameters, and provides novel perspectives for broadening the practical application.

## Graphical Abstract

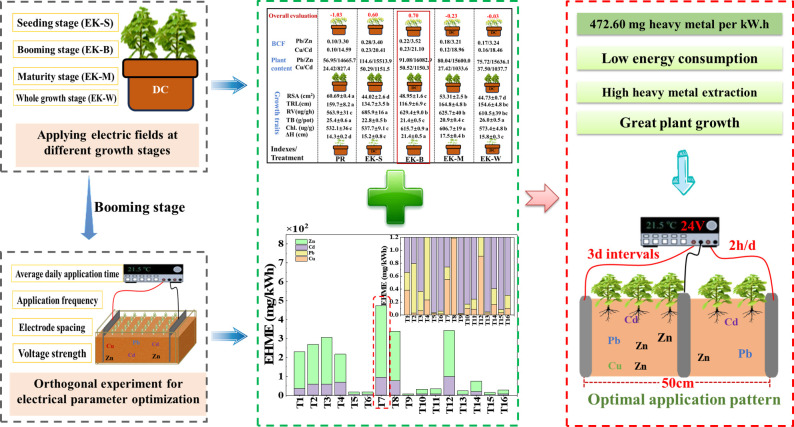

## Highlight

- Economical heavy metals extraction was studied based on plant growth stage and electrochemical parameters
- Electro-assisted of EKAPR at booming periods is alternative due to less BCF drop and higher PCA score
- Heavy metals extraction under optimal treatment higher 130.83-464.85 mg/kWh than other groups
- Regulation of plant electro-assisted growth stage and strategies contribute to more economical heavy metal extractions

## 1 Introduction

Rapid industrial development has led to various environmental problems, especially heavy metal pollution. Heavy metals accumulate in soil, water, atmospheric particles and crops and can enter the human body through direct intake, aspiration and skin contact, or indirectly through the food chain (Xie et al., 2024; Singh et al., 2025). It is worth noting that the consumption of food contaminated with heavy metals can cause irreversible damage to human health. For example, excessive exposure to Cd can seriously damage the nervous system, reproductive system, kidney function, and bone health (Sable et al., 2024). Although Zn is an essential nutrient for humans and crops, its excessive intake can lead to health risks such as anemia, cramps and neuropathy (Younas et al., 2023). In addition, heavy metal pollution leads to soil degradation, reduction of vegetation and loss of biodiversity due to its toxicity, inability to decompose, bioaccumulation and long-term presence in the environment (Xie et al., 2023). Overall, heavy metal pollution is an important component of soil pollution, which directly affects the safety of drinking water, food production and crop (Meng et al., 2024). Electrokinetic remediation is a relatively efficient *in-situ* remediation technology that has developed rapidly in recent years because it is not limited by high soil heterogeneity and low permeability, and reasonable electric field arrangement can promote the migration of heavy metals from deep soil to the surface (Gnanasundar and Akshai Raj, 2021). Combining electro-remediation with phytoremediation (electrokinetic-assisted phytoremediation technique, EKAPR) can effectively solve the problem that the effectiveness of phytoremediation is limited by the bioavailability of pollutants in the soil and the depth of plant roots (Singhal et al., 2022). In recent years, a numerous studies have demonstrated growth promotion and significant biomass increase in tobacco (0.6 V/cm), rush (1.0 V/cm), ryegrass (1.0 V/cm), and Indian mustard (1.0-2.0 V/cm) at different electric field strengths (Chen et al., 2007; Cang et al., 2012; Yuan et al., 2021). In practice, the EKAP system is usually constructed with low-intensity (< 5 V/cm) and long duration (7-60d) direct current (DC) electric field and hyperaccumulators/accumulators (rapeseed, nightshade, ryegrass, etc.). Heavy metals such as Cu, Cd, Pb, Zn, U were effectively removed (5% - 92%) from the soil after the EKAPR system treatment (Ma et al., 2024). Although electrokinetic remediation and combined technologies are effective in removing heavy metal ions from contaminated soils, the energy consumption is major obstacle for widespread application (Virkutyte et al., 2002). Therefore, it is necessary to explore an energy-saving method to make the technology feasible on engineering scale.

There are a number of factors affecting the energy consumption of electrokinetic remediation system. Previous studies have found that the material, spacing and configuration of the electrodes, and voltage can significantly affect the energy consumption of the process (Romina et al., 2021; Hamdi et al., 2024) What’s more, unreasonable application methods and long-term application of power consumption account for 10%-15% of the total cost of the electrokinetic remediation process (Jo et al., 2012; Fu et al., 2017). For example, the electromigration can be improved by increasing the voltage gradient, current intensity and electroosmotic flux, thereby promoting the efficiency of process (Cameselle, 2015). However, reverse electroosmosis may occur when uncontrolled voltage increases result in negative effects on heavy metal removal, which leads in surplus energy consumption (Wang et al., 2021). Even though researchers have found that replacing the direct-current field with a pulsed electric field (Yuan et al., 2017; Sun et al., 2015), adjusting the electrode position (Sun et al., 2019), and multi-electrode arrangement (Kim et al., 2021) can reduce the polarization potential per cycle or increase the effective electric field area to enhance the removal efficiency of heavy metals, thereby diminishing unnecessary energy consumption. In addition, the utilization of self-powered batteries such as solar cells (Jeon et al., 2015) and microbial fuel cells (Habibul et al., 2016), which produce a weak electric field through bacterial metabolism, can significantly reduce the cost of the remediation process. Wen et al. (2021) also found that the competitive properties of charged species in the soil under the electric field treatment can be enhanced by increasing the competitiveness of the target species to make them become preferred migration species, and consequently increase the efficiency of energy utilization. However, there are dual mechanisms of electric field and electrode electrochemical reaction under electric field treatment. Electric field parameters (voltage gradient, electrode spacing, frequency of electric field application and average daily application time, etc.) regulate the electric field effect, electrode electrochemical reaction and electric energy consumption of electrokinetic remediation system, and there are interactions with each other, which should be the focus of energy saving research. But there are no reports on the optimization of electric field application synergistically considering multiple electric field parameters (Yang et al., 2024). On the other hand, the actual application of the EKAPR system is mostly given to apply the electric field for a certain period of time during the process of plant growth, but too long electric field treatment time will not only damage the normal growth of the plant, but also cause energy loss (Ma et al., 2024; Sajad et al., 2020). Meanwhile, there are also differences in organ biomass distribution, plant root morphology and stress tolerance of different growth stages, which lead to differences in growth status and heavy metal accumulation ability (Zhang et al., 2021; Zhao et al., 2023). Therefore, the effect of the interaction time between electric field application and plant on EKAPR remediation process, effectiveness and energy consumption should be a critical research issue for future studies. To sum up, the study was carried out to construct a typical EKAPR pot system with one-dimensional DC commutated electric field using actual heavy metal (Cu, Cd, Pb, Zn) contaminated farmland soil and hyperaccumulater (*Sedum plumbizincicola*). Pot experiments were conducted in terms of both growth stages and electric field parameter optimization to investigate the characteristic differences between long-term and short-term electroassistance, as well as the optimal application strategy for high-efficiency accumulation coordinated with low energy consumption. Frist of all, electric field was applied to the plants at the seedling, booming, maturity and the whole growth stages. The principal component analysis (PCA) is used to comprehensively evaluate each treatment, and the optimal growth stages is selected according to the factor scores. Next, four key factors affecting energy consumption were used as control variables to design L16 (4^4^) orthogonal EKAPR pot remediation experiment. The characteristics of plant growth and heavy metal uptake under different electric field application strategies were analyzed, and the optimal application strategy was proposed based on high-efficient accumulation of heavy metals and low energy consumption of EKAPR. This study provides technical support and guidance for strengthening the mechanism of electric-assisted plant interactions, as well as the management and safe utilization of EKAPR in heavy metal-contaminated soils in agricultural fields.

## 2 Materials and methods

### 2.1 Experimental site and plant material

The plateau red soil used in the experiment was obtained from the farmland around the lead-zinc mine in Huize County, Yunnan Province. The stones, wood chips and other large impurities were removed through 3 mm screen after soil mixing the soil obtained in the field, then dried naturally and used for potting experiments of different growth stage. The pH value of 6.95, the content of organic matter, total N, P and K contents were 20.8, 1.44, 1.65 and 6.84 g/kg, and the alkali-hydrolyzale N, available P and K contents were 38.1, 71.4 and 614.3 mg/kg, respectively. The contents of Cu, Zn, Cd and Pb in soil were 302.13, 93.15, 611.00 and 6058.67 mg/kg, respectively. The content of Cu and Zn is significantly higher than the risk screening value of agricultural land (50, 200 mg/kg, 5.5 < pH ≤ 6.5), and the content of Cd and Pb is significantly higher than the risk control value of agricultural land (2, 500 mg/kg, 5.5 < pH ≤ 6.5) (GB 15618–2018 Soil Environmental Quality Risk Control Standards for Pollution of Agricultural Land). After the experiment, the remaining soil and some fresh soil was mixed for the electric field optimization experiment. The contents of Cu, Cd, Pb and Zn in soil were 247.62, 81.95, 546.00 and 5896.03mg/kg, respectively.

The polymetallic co-accumulator (*Sedum plumbizincicola*) was obtained from the farmland soil remediation base in the lead-zinc mining area of Zhehai Garden, Huize County, Yunnan Province. Healthy seedlings with uniform size were transplanted into the soil of plastic pots to carry out the experiment. No pesticides or fertilizers were applied to all plants during the whole growth periods.

### 2.2 Experiment setup

In order to investigate the characteristic differences between long-term and short-term electro-assistance of the EKAPR system and the optimization of the electric field application strategy based on the synergy of the efficient accumulation of heavy metals and energy saving, this study carried out the experiments of applying electric field treatments at different growth stages and the electric field application strategy optimization. Firstly, the growth cycle of *S. plumbizincicola* was divided into three growth stages, and the electric field treatment was applied at different growth stages and the whole growth stage to analyze the growth condition of the plants and the characteristics of the heavy metal accumulation and uptake under the long/short-term electro-assisted treatment. Thus, the optimal growth stage with the electric field application was chosen for the subsequent experiments. Secondly, the orthogonal experiments were designed with the four key factors regulating the energy consumption of the system as control variables under the optimal growth stage and phytoremediation as control. The characteristic changes of soil heavy metal removal, plant growth and plant heavy metal accumulation under different electrokinetic treatment were analyzed, and the optimal application strategy combination was proposed based on the principle of high efficiency accumulation of heavy metals and low energy consumption. The detailed experimental design is as follows:

#### 2.2.1 Electric field application at different growth stages

**Figure 1:**
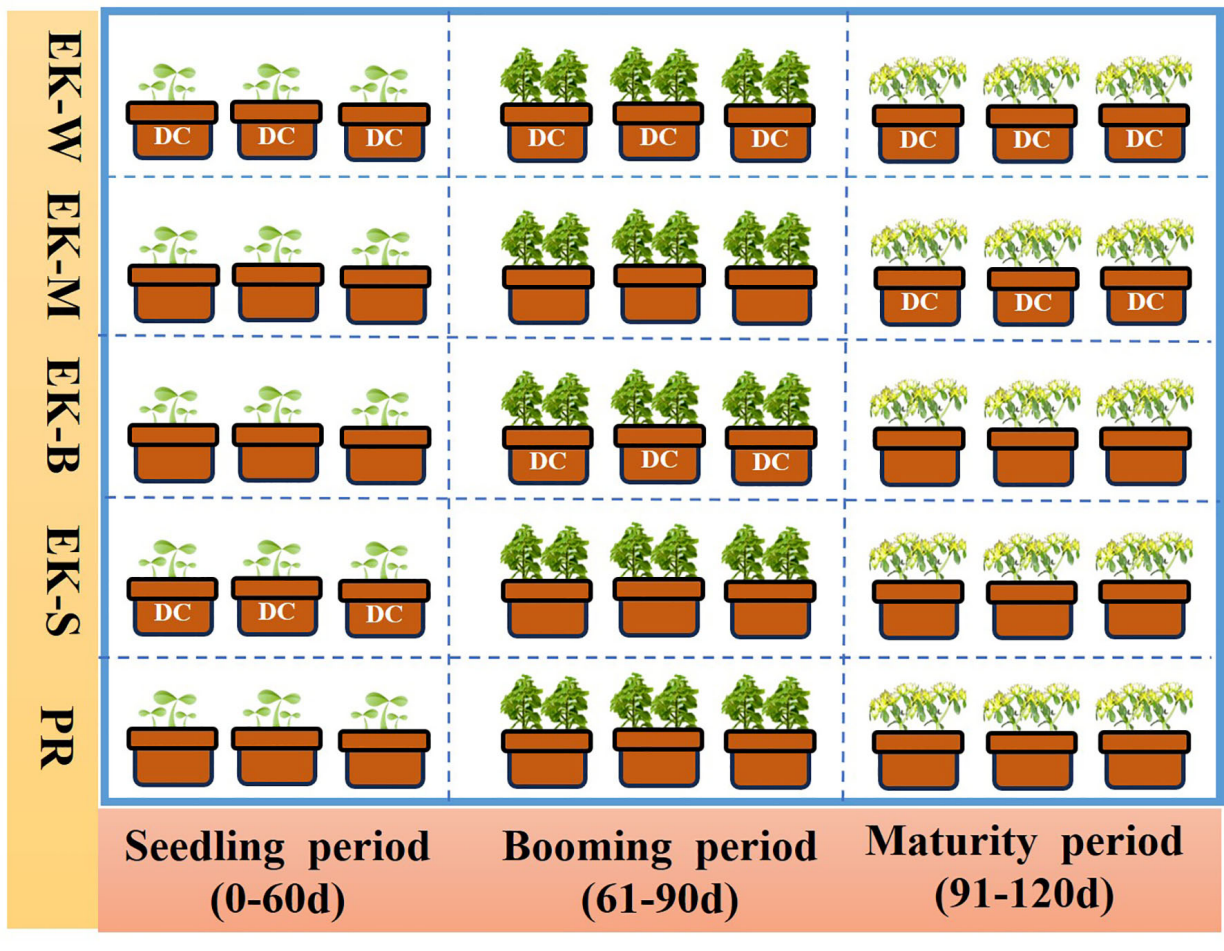
Experimental design of electric field application at different growth stages.

The proposed study was laid out in PVC rectangular pot design with triple replications, and the size of each pot was 24×15 × 17 cm. Two graphite electrodes were inserted vertically into the soil on both sides of the pot, and a direct current power supply (0–100 V, 0–3 A) as the output power. Electric field treatments were applied to the plants at seedling (0-30d, EK-S), booming (31-60d, EK-B), maturity (61-90d, EK-M) and whole growth periods (0-90d, EK-S), respectively. And no electric field treatment during the whole growth stage as a control (PR). The schematic of the experimental design was shown in **Figure 1**. All treatments were sampled at 90 days after transplanting. The voltage of 1V/cm electric field were conducted between 8 a.m. and 12 a.m. in every treatment. All other agronomic practices were done uniformly for all treatments.

#### 2.2.2 Orthogonal experimental of different electric field application strategies

Orthogonal experimental design is a statistical method suitable for multi-factor experiments, using orthogonal tables to study the influence of multiple factors and levels on the test results (Feng et al., 2022; Wang and Ding, 2022). Orthogonality can be used to obtain the required data properly and efficiently, and analyze the influence of each parameter to get the best combination. In this study, the electric field parameters such as voltage gradient (A), electrode spacing (B), electric field application frequency (C) and average daily application time (D), affecting energy consumption, were used as control variables to carry out orthogonal pot experiments of at the optimal growth stage. Each factor includes four levels, the values chosen for each level were based on the appropriate range found in the literature. The was used as control (CK) and the experimental design is shown in **Table 1**. The EKAPR pots were made of PVC material in a rectangular culture chamber with the dimensions of L×W×H=57×32×18 cm (**Supplementary Figure S1**). The pot experiments were carried out 4 cycles, with the electric field reversal every 7 days recorded as one cycle, and three replications were set for each treatment. The current was recorded dynamically at regular intervals, and soil and plant samples were collected at the end of experimental for the determination.

**Table 1:** Levels and factors of the L16 (4^4^) orthogonal experimental design.

Treatments	A Electric field strength (V)	B Electrode spacing (cm)	C Frequency of electric field applied (intervals)	D Application time (h/d)
T0 (CK)	–	–	–	–
T1	12	12.5	0	2
T2	12	16.7	1	4
T3	12	25.0	2	8
T4	12	50.0	3	12
T5	24	12.5	1	8
T6	24	16.7	0	12
T7	24	25.0	3	2
T8	24	50.0	2	4
T9	36	12.5	2	12
T10	36	16.7	3	8
T11	36	25.0	0	4
T12	36	50.0	1	2
T13	48	12.5	3	4
T14	48	16.7	2	2
T15	48	25.0	1	12
T16	48	50.0	0	8

### 2.3 Data collection and sample analysis

At the experiment, the fresh plants were washed with tap water to remove the adherent soil and then cleaned three times with pure water. And the plants were divided into shoots (aboveground parts) and roots. Fresh samples were used to determine total chlorophyll content, root vigor and root morphology. After a half hour of water removal at 105°C, the samples were dried at 75°C in an oven, and the biomass and heavy metals contents were determined.

#### 2.3.1 Plant growths and physiological analysis

Plant growth parameters relate to plant height, dry weight, chlorophyll content, root vigor and root morphology. Plant height increase (ΔH) was obtained by the difference of plant height between planting (0d) and harvesting (90d). The plant height and dry weight were measured with ruler and balance. Roots were scanned using an EPSONV700 flatbed scanner (Seiko Epson, Japan) and then the root morphology parameters were analyzed using Win RHIZOPROSTD4800 type (Regent, Canada) root image processing software. The chlorophyll content was extracted by ethanol-acetone method. The root vigor was qualitatively determined by triphenyltetrazolium chloride (TTC) method, and the reduction strength of tetrazolium per unit fresh root weight was used to represented the root vigor.

#### 2.3.2 Phytoextraction efficiency

Heavy metal uptake indicators include heavy metal accumulation in plants, translocation factor (TF) and bioconcentration factor (BCF). Soil samples were digested with aqua regia in the electric hot plate until the solution is becoming colorless and transparent. The plant samples were placed in polytetrachloroethylene tank with 3mL concentrated nitric acid and 3mL 30% H_2_O_2_ solution, and heated in a constant temperature drying oven (120-160°C) for 4h. Then, the sample was filtered with purified water into 50 mL volumetric flask to be calibrated, and the Cu, Zn, Cd, and Pb contents in the soil and plant were determined by flame atomic absorption spectrophotometer. And the heavy metal accumulation, BCF and TF were used to evaluate plant phytoextraction efficiency, and were calculated by the following equations:


Heavy metal accumulation=Heavy metal content in plant×biomass

Shoot(Root)BCF=Heavy metal content in shoot(root)/total soil heavy metal content

TF=Heavy metal content in shoot/heavy metal content in root

In addition, the extraction of heavy metals per unit of energy consumption was chosen to evaluate the treatment effect of different electric field application strategies in order to select the optimal electric field application strategy with high heavy metal accumulation and low energy consumption, and the calculation formula was as follow:


EC=U×I×t

$$\text{EHME}(\text{mg}/\text{kWh})=\frac{\text{Plant heavy metal accumulation increase }}{\text{Total energy consumption }}\;$$

Where, EC = energy consumption (kWh), U = Voltage between the two electrode plates (V), I = average electric current of EKAPR system (A), EHME= Economical heavy metals extraction.

#### 2.3.3 Correlation and Principal component analysis

Pearson correlation coefficient was used for correlation analysis and principal component analysis (PCA) was used for comprehensive evaluation of each treatment. And PCA and correlation plots of optimization experiments were employed for investigating the multiple relationships between the plant growths and heavy metals content in plant with energy consumption. Plant growth indexes were selected related to heavy metal accumulation, such as plant height, total dry weight, root vigor, total root length, total root surface area, net photosynthetic rate and total chlorophyll content. Heavy metal indicators were selected for the average content of Cu, Zn, Cd and Pb from the plants.

#### 2.3.4 Range analysis of orthogonal experiments

The effects of electric field strength, electrode spacing, electric field application frequency and daily application time on heavy metal extraction per unit energy consumption were analyzed by range analysis. The range (R) is the difference between the mean of the maximum and minimum values for each factor level, and the effect of each level on the factor can be determined by the range of levels. When R is large, the factor level causes significant changes in the response data, indicating that the factor has a significant impact on the response value (Peng et al., 2020). Therefore, the importance of these factors can be ranked using the R-value. K1, K2, K3, and K4 represent the average of Level 1, Level 2, Level 3 and Level 4 (daily application time), respectively. The optimal condition is determined by the maximum value of K (K_max_) in each factor.

### 2.4 Statistical analysis

Statistical analysis and graphing were performed with Origin 2024 and IBM SPSS 25.0. One-way analysis of variance (ANOVA) and Duncan test were used to determine differences between treatments. To ensure the accuracy of experimental data, *p* < 0.05 at the probability level is statistically significant, and all experimental data were averaged with three repetitions.

## 3 Results

### 3.1 Effect of electrokinetic application on *S. plumbizincicola* at different growth stages

**Figure 2:**
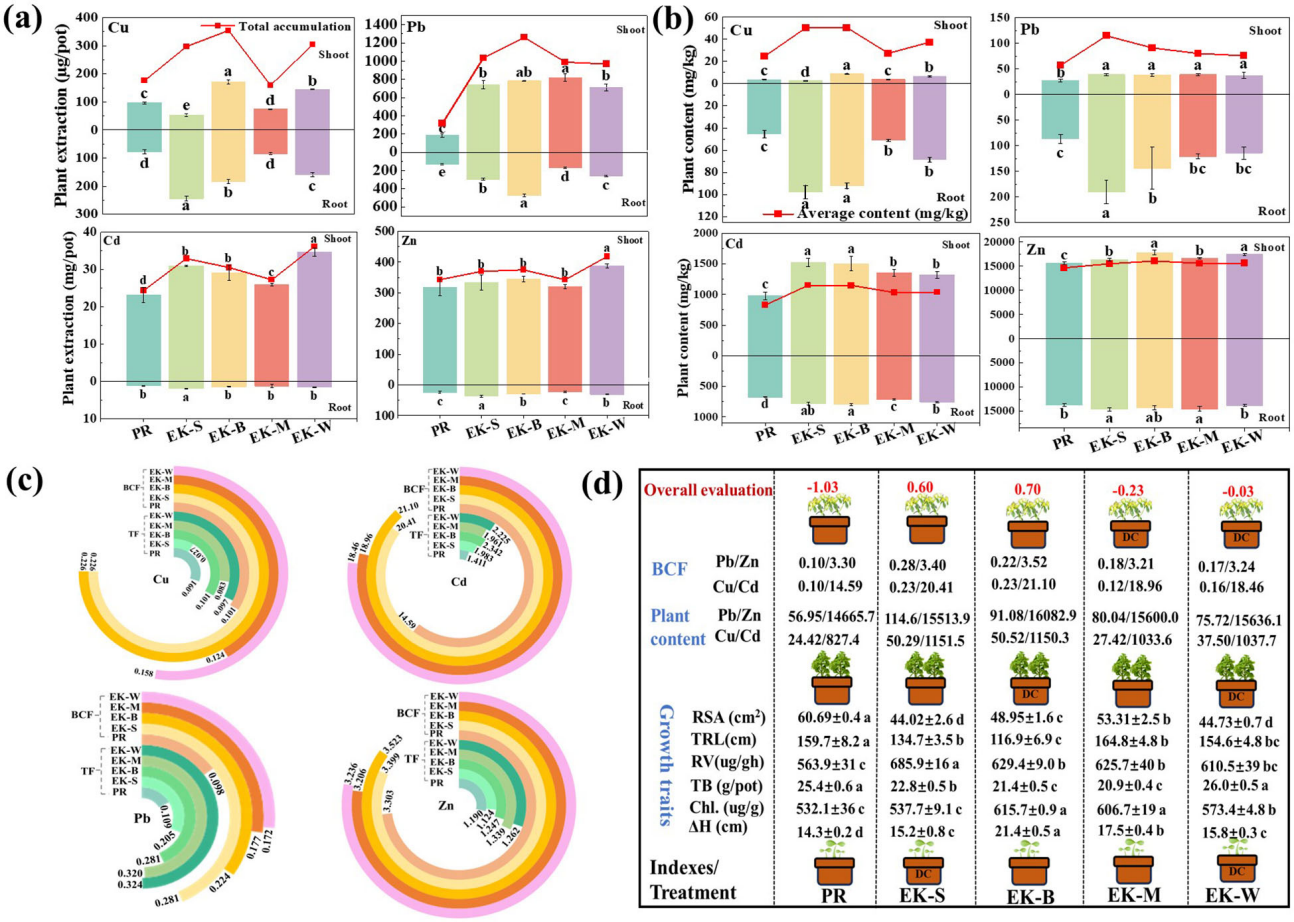
Effects difference of electric field application at different growth stages on extraction **(a)** and content **(b)** of heavy metal in plant, BCF and TF **(c)**, and plant growth traits **(d)**. (ΔH, plant height; TRL, Total Root Length; RSA, Surface Area; RV, Root Vigor; TB, Total biomass; Chl., Chlorophyll content).

The application of electrokinetic at different stages will also have different effects on *S. plumbizincicola* because of the significant differences in nutrient uptake, water use and photosynthesis and growth characteristics at different growth stages. In this study, the differences in the extraction, contents, TF and BCF of heavy metal by *S. plumbizincicola*, as well as its growth characteristics after applying electric field treatments at different growth stages were investigated. As can be seen in **Figures 2a, b**, the application of electrokinetic treatment significantly promoted the heavy metals accumulation in *S. plumbizincicola* compared to phytoremediation alone (PR treatment), especially the extraction. The average content did not increase with the increase of electric field application time whatever of hyperaccumulation (Cd, Zn) and non-enriched (Cu, Pb) species, instead they were higher under the EK-S and EK-B treatments. In terms of heavy metal content, the difference in the effect of electric field application at different growth stages on Cu and Pb in the roots was more obvious, whereas Zn and Cd showed more significantly in the aboveground. Overall, it was found that electric application at seedling stage and booming stage was more desirable for increasing the content of four heavy metals in *S. plumbizincicola*, especially for Cu and Pb. The total extraction of Cu and Pb showed similar trends with the content change, and maximum in EK-B which were 101.87% and 298.98% higher than PR treatment, respectively, followed by EK-W, EK-S and EK-M. However, the Cd and Zn showed different trends with the content change, and in the order of EK-W (36.17, 417.93 mg/pot) > EK-B (30.51, 374.70 mg/pot) ≈ EK-S (32.91, 369.75 mg/pot) > EK-M (27.21, 342.21 mg/pot). Compared with PR treatment, it increased by 105.93% and 106.86%, 39.17% and 39.02%, 101.30% and 59.94%, 5.78% and 9.66%, respectively.

In addition, BCF and TF showed a significant increase in all treatments after applying the electric field compared to the PR treatment. Among the four heavy metals, BCF increased significantly under EK-S (Cu: +123.76%, Cd: +39.89%, Pb: +186.73%, Zn:+2.91%) and EK-B (Cu: +123.76%, Cd: +44.62%, Pb: +128.57%, Zn:+6.66%) treatments, while TF increased significantly under EK-W (Cu: +6.59%, Cd: +57.69%, Pb: +197.25%, Zn:+6.05%) treatment obviously, and followed by EK-M, EK-B and EK-S. The variation of BCF and TF showed similar trends to those of heavy metal contents and total extractions in plants. As shown in **Figure 2d**, the application of electric field significantly increased ΔH, chlorophyll content and root vigor of *S. plumbizincicola*, whereas significantly inhibited plant biomass (except for EK-W). Comprehensive evaluation combining plant growth characteristics and heavy metal extraction revealed that the order of factor scores was EK-B (0.70) > EK-S (0.60) > EK-W (-0.03) > EK-M (-0.23) >PR (-1.03). These results indicate that the application of electric field treatment during a specific growth stage can effectively replace whole growth stages to attain soil heavy metal remediation efficiently and achieve better energy saving. Furthermore, the best results were obtained by applying the electric field treatment during the blooming stage, and the subsequent studies will be conducted under this treatment as well.

### 3.2 Optimization analysis of electric field parameters

#### 3.2.1 Plant growth characteristics

Based on the study in section 3.1, electric field treatment was applied during the booming stage of *S. plumbizincicola*, and orthogonal potting experiments were designed to investigate the effects of voltage intensity, electrode spacing, electric field application frequency and average daily electric field application time on plant growth conditions. From **Table 2**, it can be seen that the plant indexes of *S. plumbizincicola* were significantly increased compared to CK, except that the ΔH and net photosynthesis rate showed significant inhibition under some treatments (such as T9). The factors of electric field parameters significantly (*p* ≤ 0.05) impacted the plant heigh and root morphology such as root surface area and root volume, but did not significantly (*p* ≥ 0.05) impact on total dry weight, net photosynthetic rate, total chlorophyll content and root vigor (**Supplementary Table S1**). The maximum ΔH, total dry weigh and root vigor were related to lager voltage (36V or 48V), and the largest values of them were 67.57% (T10), 61.54% (T10) and 304.88% (T11) higher than the control group, respectively (**Table 2**). And the greatest total chlorophyll content, total root length, total root surface area and root volume were related to plants treated by lower voltage (12V). Meanwhile, the **Supplementary Table S2** results also showed that large electrode spacing and slow application frequency have the greatest influence on all growth traits, except total dry weight (small electrode spacing and quick application frequency). For average daily application time, the optimum level of plant growth observed in 4h/d treatment, but not total dry weight (12h/d treatment). Nevertheless, the growth characteristics of *S. plumbizincicola* did not show obvious trend with the factors, probably due to the synergistic influence of multiple factors.

**Table 2:** Plant growth characteristics difference under orthogonal experiment treatment.

Treatments	Factors				ΔH (cm)	Total dry weight (g/pot)	Net photosynthetic rate	Chlorophyll content (μg/g)	Root vigor (ug/(g.h))	Total root length (cm)	Total root surface area (cm^2^)	Root volume (cm^3^)
	A	B	C	D								
T0	CK				3.70 ± 0.53^d^	8.06 ± 0.17^i^	4.02 ± 0.71^ef^	400.6 ± 26.6^j^	233.3 ± 41.6^g^	30.31 ± 6.73^hi^	5.64 ± 1.08^hi^	0.22 ± 0.03^efg^
T1	12	12.5	0	2	3.16 ± 0.05^e^	12.20 ± 0.10^abc^	3.31 ± 0.24^fgh^	723.7 ± 81.6^ef^	375.3 ± 69.6^ef^	57.20 ± 9.45^ef^	7.38 ± 0.62^fg^	0.34 ± 0.12^bc^
T2	12	16.7	1	4	5.04 ± 0.19^b^	10.65 ± 0.18^fg^	2.79 ± 0.51^ghi^	705.2 ± 44.9^ef^	219.9 ± 47.4^g^	92.96 ± 4.90^c^	8.85 ± 0.73^def^	0.54 ± 0.03^a^
T3	12	25.0	2	8	3.15 ± 0.45^e^	10.78 ± 0.83^fg^	10.5 ± 1.63^b^	803.7 ± 8.51^cd^	103.2 ± 5.37^h^	50.25 ± 2.57^fg^	8.48 ± 0.37^ef^	0.39 ± 0.01^b^
T4	12	50.0	3	12	2.27 ± 0.17^f^	9.93 ± 0.21^gh^	6.28 ± 0.36^d^	930.9 ± 57.1^ab^	236.8 ± 29.1^g^	125.2 ± 10.2^b^	11.3 ± 2.00^b^	0.52 ± 0.10^a^
T5	24	12.5	1	8	2.80 ± 0.20^ef^	11.22 ± 0.10^def^	7.48 ± 1.14^c^	734.5 ± 43.6^de^	352.6 ± 50.0^f^	29.44 ± 1.91^i^	4.55 ± 0.24^i^	0.16 ± 0.04^g^
T6	24	16.7	0	12	1.22 ± 0.18^g^	12.83 ± 0.12^a^	13.4 ± 0.67^a^	586.1 ± 44.8^hi^	236.7 ± 27.5^g^	66.71 ± 5.39^de^	7.93 ± 0.39^fg^	0.32 ± 0.05^bcde^
T7	24	25.0	3	2	3.12 ± 0.24^e^	10.57 ± 0.81^fg^	4.45 ± 0.56^ef^	959.1 ± 36.9^a^	430.2 ± 23.2^e^	69.84 ± 4.03^de^	7.31 ± 0.45^fg^	0.24 ± 0.10^defg^
T8	24	50.0	2	4	4.07 ± 0.12^cd^	9.28 ± 0.67^h^	1.39 ± 0.44^j^	646.2 ± 49.2^fghi^	822.2 ± 22.3^b^	150.9 ± 17.2^a^	13.8 ± 0.38^a^	0.37 ± 0.06^bc^
T9	36	12.5	2	12	1.63 ± 0.20^g^	11.78 ± 0.35^bcde^	1.72 ± 0.21^ij^	697.7 ± 33.9^efg^	503.5 ± 31.6^d^	42.43 ± 4.02^gh^	6.45 ± 0.76^gh^	0.16 ± 0.04^g^
T10	36	16.7	3	8	6.20 ± 0.20^a^	13.02 ± 0.10^a^	4.64 ± 0.83^e^	584.5 ± 20.2^i^	424.5 ± 23.7^e^	69.43 ± 5.07^de^	7.83 ± 0.45^fg^	0.27 ± 0.02^cdef^
T11	36	25.0	0	4	4.09 ± 0.40^cd^	12.18 ± 0.75^abcd^	7.87 ± 0.99^c^	872.5 ± 36.3^bc^	944.6 ± 18.5^a^	76.63 ± 13.0^d^	9.55 ± 1.34^cde^	0.19 ± 0.02^fg^
T12	36	50.0	1	2	6.66 ± 0.75^a^	10.31 ± 0.55^fg^	2.14 ± 0.18^hij^	620.4 ± 18.8^ghi^	431.8 ± 61.3^e^	71.67 ± 0.85^d^	9.62 ± 0.60^cde^	0.34 ± 0.01^bcd^
T13	48	12.5	3	4	4.10 ± 0.20^cd^	11.28 ± 0.63^cdef^	1.98 ± 0.24^ij^	826.4 ± 48.7^c^	332.7 ± 34.1^f^	97.82 ± 1.86^c^	10.1 ± 0.11^bcd^	0.29 ± 0.04^cdef^
T14	48	16.7	2	2	4.60 ± 0.19^bc^	12.47 ± 0.88^ab^	2.76 ± 0.34^ghi^	665.1 ± 49.6^efgh^	598.4 ± 70.0^c^	92.62 ± 8.20^c^	8.51 ± 0.60^ef^	0.28 ± 0.03^cdef^
T15	48	25.0	1	12	2.33 ± 0.21^f^	12.70 ± 0.68^ab^	4.32 ± 0.34^ef^	646.2 ± 52.4^fghi^	813.3 ± 30.7^b^	44.45 ± 6.61^fg^	6.52 ± 0.61^gh^	0.20 ± 0.02^fg^
T16	48	50.0	0	8	4.26 ± 0.12^cd^	10.95 ± 0.50^ef^	3.75 ± 0.20^efg^	719.0 ± 19.1^ef^	103.3 ± 9.65^h^	90.47 ± 8.96^c^	10.7 ± 1.05^bc^	0.35 ± 0.05^bc^

A, B, C, D represents the four influences (electric field strength, electrode spacing, electric field application frequency and average daily application time), and different lower letters in each row in table indicate significant differences between the treatments (*p*<0.05).

#### 3.2.2 Heavy metal accumulation difference

**Figure 3:**
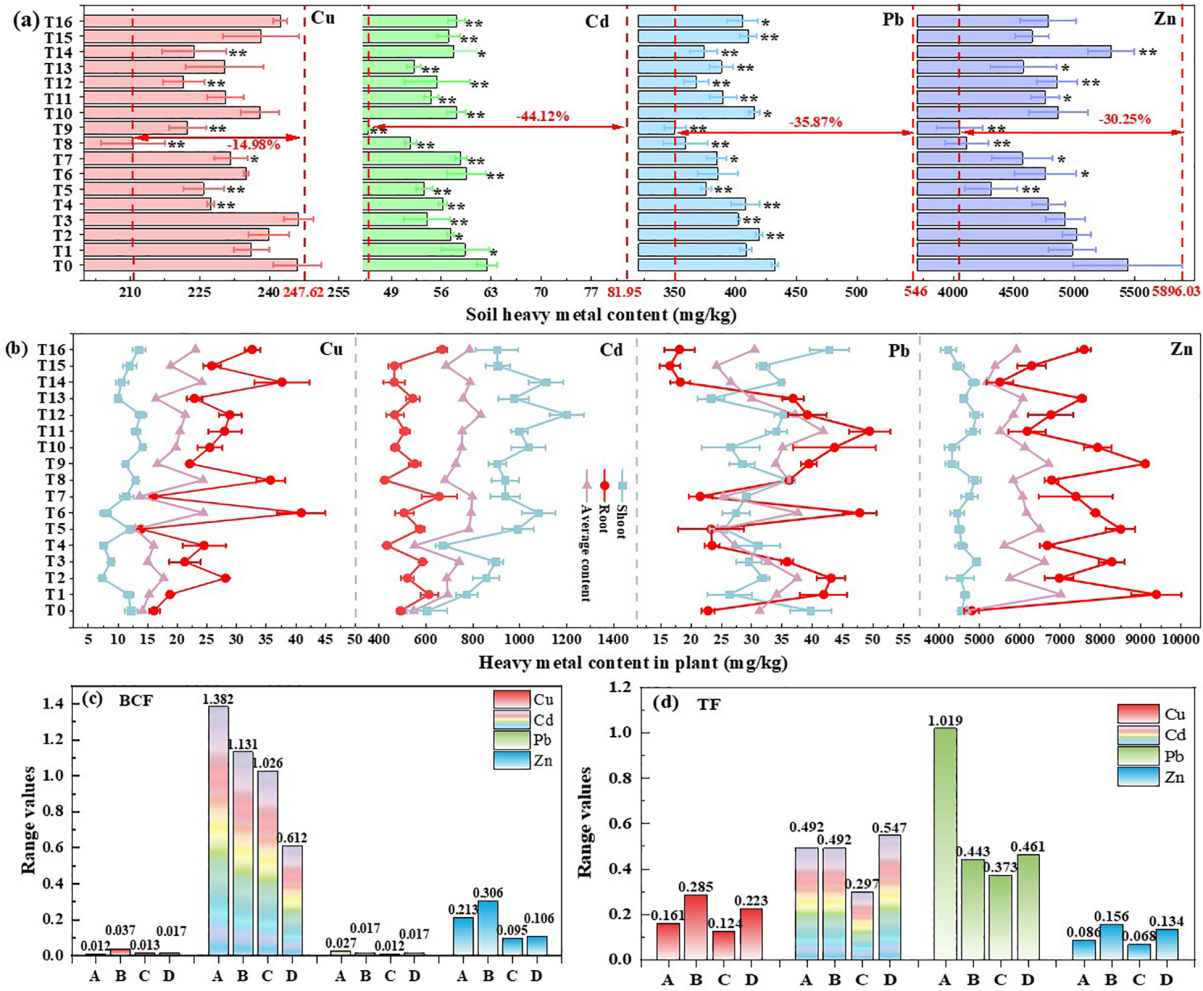
Effects difference of different electric field application on heavy metal content of soil **(a)** and plant **(b)**, the range values of BCF **(c)** and TF **(d)**. *(*p*<0.05), **(*p*<0.01) indicates the significance electric field treatment with respect to the T0 treatments.

In order to explore the removal effectiveness of heavy metals by *S. plumbizincicola* under different electric field application strategies, the heavy metal contents in soil and plants after pot treatments were determined, respectively. Compared with the original soil, the contents of all four heavy metals in the soil after potting treatment exhibited significant reductions. The maximum removal of Cu, Cd, Pb and Zn was 14.98% (T8), 44.12% (T9), 35.87% (T9) and 30.25% (T9), respectively. After the electric field treatment, soil heavy metals contents were further reduced, especially the Cd and Pb. Compared with T0 treatment, the heavy metals content showed extremely significant decreasing trend under different electric field treatments (**Figure 3a**). And the content of Cu and Zn reached lower values under the T8 and T9 treatments, which were 14.43%, 24.46% and 9.63%, 25.57% lower than control group, respectively. **Figure 3b** reveals a notable increase in the average content of heavy metals in the plants after electric field treatment, especially the content of Cd (+24.23%-52.09%) and Zn (+10.81%-50.11%) in plants. Also, the higher plant content of Cu, Cd and Pb related to treated by a high voltage strength (36 or 48V), and smaller electrode spacing (16.7 cm), application frequency (0 interval) and average daily application time (2 or 4 h/d), the maximum average contents were 24.40 (T4), 834.13 (T12) and 42.24 (T4) mg/kg, respectively. But the Zn content related to treated by a low voltage strength (12V), electrode spacing (12.5 cm) and application frequency (0 interval), and long average daily application time (8 h/d), the greatest Zn contents were observed in T1 treatment (7017.48 mg/kg). In order to investigate the effects of different factors on the heavy metal accumulation in plant, the range values of BCF and TF were calculated. The range value of Cd and Zn is significantly larger than that of Cu and Pb due to their larger BCF value. From **Figure 3c**, it can be found that the most influential factor on the enrichment of Cu (B>D>C>A) and Zn (B>A>D>C) is the electrode spacing, and that of Cd (A>B>C>D) and Pb (B>A>D>C) is the voltage strength. The TF value of Cu, Cd and Pb is significantly larger than that of Zn. The influence of factor A and B on the TF values of Cu and Zn was significantly greater than that of factor C and D, frequency of electric field application (C) had the weakest effect on the TF value of Cd, the greatest effect of voltage strength (A) on Pb, and insignificant variations among the remaining factors (**Figure 3d**).

### 3.3 Parameter optimization design based on orthogonal experiments

**Figure 4:**
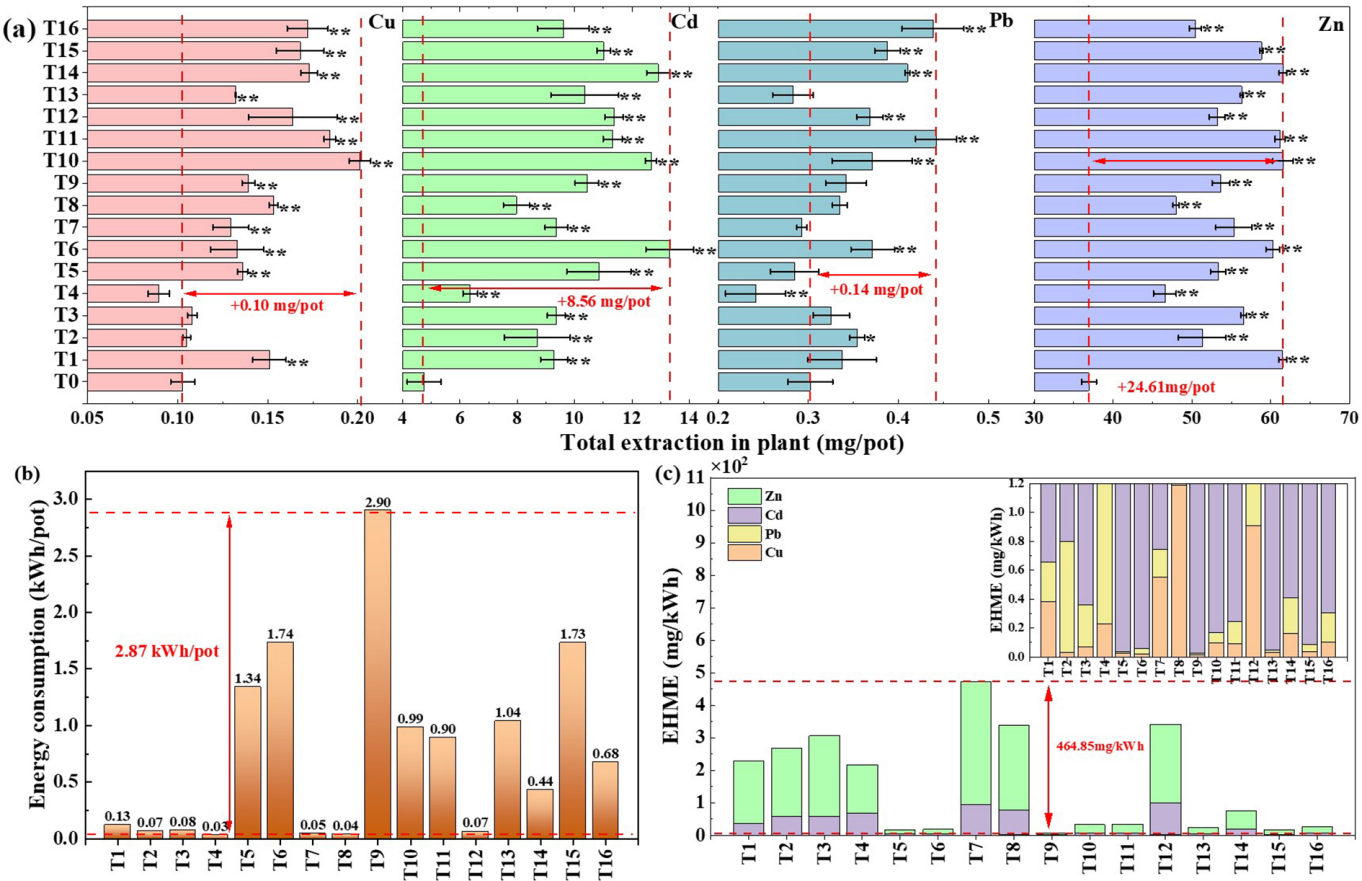
Effects difference of different electric field application on heavy metal extraction **(a)** energy consumption **(b)** and EHME **(c)**. *(*p*<0.05), **(*p*<0.01) indicates the significance electric field treatment with respect to the T0 treatments.

The effect of electric field parameters on heavy metal extraction, energy consumption and the economic heavy metal extraction (EHME) were shown in **Figure 4**. The extraction of Cu, Cd, Pb and Zn from plants after electric field treatment was significantly increased by 2.08%-100.44%, 47.49%-180.00%, 6.68%-46.29% and 4.17%-104.49% over the T0 treatment, and its maximum extraction increased by 0.10 (T10), 8.56 (T6), 0.14 (T11) and 24.61 mg/pot (T11), respectively (**Figure 4a**). The results also showed that 36 or 48V voltage, 25cm electrode spacing, 2d interval and 4h/d treatment had the greater average extraction, and voltage had the greatest influence on extraction of Cu and Pb, as well as electrode spacing had the greatest influence on extraction of Cd and Zn (**Supplementary Table S4**). The energy consumption of the EKAPR system under different electric field parameter treatments varied significantly ranging from 0.03 to 2.90 kWh (**Figure 4b**). Electric field strength, electrode spacing and average daily application time significantly impacted energy consumption, the smallest values were observed in 12V voltage, 50cm electrode spacing, 3d interval and 12h/d treatment (T4).

Based on the principle of low energy consumption and high heavy metal extraction, the heavy metal extraction increment per unit of energy consumption (EHME) was calculated (**Figure 4c**). The largest EHME value were obtained in T7 treatment (472.60 mg/kWh), which were130.83-464.85 mg/kWh higher than other groups. Secondly, T3 (306.64 mg/kWh), T8 (337.92 mg/kWh) and T12 (341.77 mg/kWh) treatments also showed higher heavy metal extraction increments. In addition, the EHME increased with electrode spacing (K4>K3>K2>K1), and decreased voltage (K1>K2>K3>K4), application frequency (K4>K3>K2>K1) and average daily application time (K1>K2>K3>K4), but variance analysis results showed that the four factors all insignificantly impacted the EHME in EKAPR system (**Supplementary Table S5**). According to the range analysis, electric field strength had the greatest influence on EHME, followed by average daily application time, electrode spacing and application frequency, and the optimal combination is A1B4C4D1 (voltage strength of 12 V, electrode spacing of 50 cm, and interval of 3 d for 2 h/d) for the *S. plumbizincicola* in EKAPR system.

## 4 Discussion

### 4.1 Electro-assisted at booming stage can replace long-term electric field application

Previously, the EKAPR system has been widely used for the removal of heavy metals and organic pollutants from soils due to its ability to alleviate the water-soluble ion decay and maintain the soil colloidal properties (Gao et al., 2021), as well as to stabilize the soil pH by plants and solve the problem of acidification of the soil after electric remediation (Osman et al., 2022), and to promote the dissociation of the effective nutrients and heavy metal ions in soil (Ma et al., 2022). Especially, electroosmosis and electromigration in the electrokinetic action can effectively promote the available form of heavy metal and migrate with the direction of electric field, so as to effectively improve the remediation efficiency of the EKAPR system for heavy metal. After EKAPR system treatment, soil heavy metals such as Cu, Cd, Pb, Zn and U can be effectively removed (removal rates from 5% -92%) (Ma et al., 2024). On the other hand, electrokinetic assistance with appropriate intensity (0.5-4.0 V/cm) can stimulate cell division, enhance enzyme activity, and increase chlorophyll synthesis, thereby promoting plant growth (Li et al., 2016; Zhao et al., 2020). In the study, the electric field application with 1 V/cm at the seedling, blooming and mature stages and whole stage of *S. plumbizincicola* found that the phytoextraction, content in the plant and BCF of Cu, Cd, Pb, and Zn under all treatments showed an increasing trend compared to the absence of the electric field (PR treatment). And there was slight increasing of plant growth, which were similar to the results of the previous study. Interestingly, the application of electric field treatments at individual growth stages (EK-S, EK-B, EK-M) and the whole growth stage (EK-W) achieved effective extraction of heavy metals by *S. plumbizincicola*. Even the average content of the four heavy metals in the *S. plumbizincicola* under the EK-S and EK-M treatments were slightly higher than that under the EK-W treatment (**Figure 2b**). This is consistent with the findings of Sajad et al. (2020) that the removal of soil contaminants did not significantly increase with increasing remediation time (40% for 7d and 42% for 10d), and that even too long electrokinetic remediation time results in unnecessary power consumption (Han et al., 2014). Moreover, even with the application of low electric field intensity, too long treatment time is still unfavorable for plant growth (Xu et al., 2020).

Nevertheless, there were still differences in heavy metal uptake and plant growth characteristics of *S. plumbizincicola* after electric field treatment at different growth stages. The differences in the effects of electric field on different growth stages can be summarized as follows: First, differences in photosynthetic rates. With the plant growth, there are significant structural, chemical and functional changes in the leaves at different ages that alter the distribution and uptake processes of nutrients (Warren, 2006). Additionally, leaf specific gravity, leaf nitrogen content and water use efficiency increased with growth, but photosynthetic nitrogen use efficiency and stomatal conductance decreased (Renninger et al., 2015), thus photosynthetic rate showed significant variations among growth stages. For example, it was found that the photosynthetic rate, intercellular CO_2_ and N contents of cotton were the highest at the bud stage, followed by the flowering stage (Shah et al., 2021). Our study also found that the total chlorophyll content in the leaves of *S. plumbizincicola* showed an increasing trend with increasing growth time under the electric field treatment, reaching a maximum in the booming stage (EK-B treatment). Second, differences in nutrient absorption. It has been shown that nitrogen and phosphorus content in plants tends to show large differences at different growth stages due to different tissue functions (Li et al., 2014). Compared with the seedling stage, more nitrogen is utilized for photosynthesis, and the synthesis of proteins and nucleic acids to meet plant growth needs in the later growth stages, and therefore has a higher uptake of nitrogen and phosphorus (Goldberg et al., 2017; Iles et al., 2016). Since electric field treatment can effectively dissociate the active nutrients in the soil, a large amount of active nutrients was dissociated from the soil under the EK-W treatment, resulting in the biomass of *S. plumbizincicola* under this treatment being significantly higher than that under other treatments (+2.36%-24.40%). Whereas the reduction in biomass under the other electric field treatments may be attributed to the stress effect of the electric field (Xu et al., 2020). Third, differences in heavy metal uptake and accumulation. The uptake of heavy metals by plants is a complex physical, chemical and biological process, which is influenced by the plant type, growth stage, etc. Promotion of nutrient and non-nutrient metal uptake from soil with the help of membrane transporter proteins during plant growth (Giovanni et al., 2013). The rhizosphere microbial community, which affects the accumulation of heavy metals in plants, also changes with plant growth, in line with the needs of the plants (Qin et al., 2024). Our study found that the lowest accumulation of the four heavy metals by the *S. plumbizincicola* was EK-M treatment, while the remaining three treatments did not differ significantly. The low accumulation of heavy metals under EK-M treatment may be due to low biomass (**Figure 2d**) on the one hand, and may be related to the rhizosphere soil microbial community on the other hand (Farina et al., 2012). Fourth, differences in plant stress capacity. As the plant grows, the oligofructose level in the plant decreases and the sucrose, glucose and fructose content increases, leading to a gradual increase in the antioxidant activity of the plant (Arena et al., 2024; Zou et al., 2021). Debojyoti et al. (2023) found that the sensitivity of rice to arsenic stress was in the order of flowering > grouting > maximum tillering. Compared with PR treatment, the biomass of *S. plumbizincicola* decreased significantly due to the stress of electric field, and the biomass decreased most obviously at maturity stage, which may be due to the poor adaptability of plants to environmental changes at maturity stage (Gao et al., 2019). In conclusion, the application of electric field in a single growth period can effectively replace the long-term application of electric field and save as much power loss as possible. Furthermore, the comprehensive evaluation of *S. plumbizincicola* is better after applying electric field treatment in the booming period.

### 4.2 Optimizing electric field parameters improves plant extraction of heavy metals

The EKAPR system is a complex ecosystem composed of external electric field, remediation plants and soil medium, and its influence factors are complicated and diverse. Among them, electrokinetic parameters such as electrode type, electrode spacing, electrode configuration, voltage intensity and electric field application time affect the remediation efficiency of the EKAPR system on the one hand, and are closely related to the electrical energy loss on the other hand (Hamdi et al., 2024; Ghobadi et al., 2021). In this study, the orthogonal pot experiment found that the heavy metals extraction in plants increased significantly under different electric field application strategies (**Figure 3a**). The reasons may be attributed to the following: First of all, suitable electric field can improve the permeability of plant cell membrane to heavy metal ions to biofilm, thus increasing the heavy metals content in *S. plumbizincicola* (Putra et al., 2013). Secondly, the internal electric field in the plant prevents the diffusion of charged ions in the plant tissue, and the applied electric field can balance this phenomenon (Raghavan et al., 2013). Thirdly, electroosmosis by direct current electric field promotes the migration of dissolved cations to the negatively charged plant roots (Cameselle and Reddy, 2012). Furthermore, the application of electric field can also promote plant roots to secrete low molecular organic acids, which activates heavy metals or forms soluble complexes, and enhances the absorption of heavy metals by the plant root system (Hu et al., 2020).

**Figure 5:**
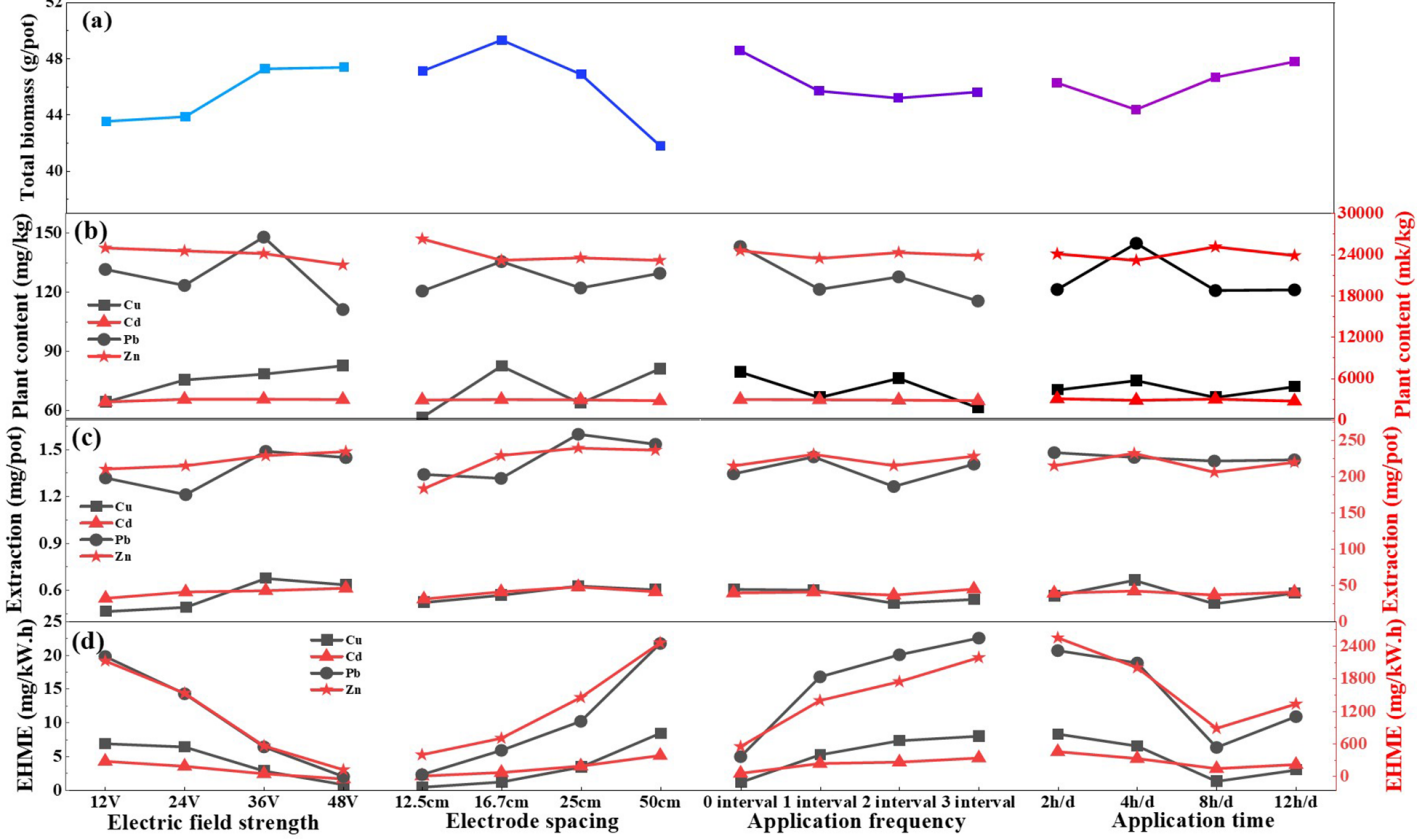
Effects difference of different electric field application on factor level diagram of biomass **(a)**, heavy metal in plant **(b)**, plant extraction **(c)** and EHME **(d)**.

As early as 2011, the study of Cang et al. (2011) found that the electric field gradient is one of the most important factors affecting plant growth, soil properties and soil mineral concentration. Low voltage gradients are usually applied in EKAPR potting experiments because higher voltage gradients can lead to loss of physicochemical properties of the soil (Sánchez et al., 2020), as well as jeopardize the normal plant growth process (Fan et al., 2021). Compared with the PR treatment in this study, all the growth indicators of *S. plumbizincicola* under electric field treatment showed an increasing trend (**Table 2**), and the biomass increased with voltages and decreased with electrode spacing (**Figure 5a**). The smaller the electrode spacing, the higher the current (Wen and Yan, 2020), and the greater the stress on the plant, which is not beneficial to plant growth. The continuous application of electric field has a better promoting effect on biomass than the interval application, but the daily application time only exhibited a slight promoting effect (**Figure 5a**). Putra et al. (2013) also found that under the electric field treatment at 50 Hz, the content of chlorophyll a and chlorophyll b in Kentucky bluegrass increased by 17% and 44%, compared with that at 10 Hz, respectively. In terms of the content and extraction of heavy metal in plants, the differences between the factors were not significant, even though electric assistances played a contributing role with the extraction higher 7.20-32.94 mg/pot than PR treatment (**Figures 5b, c**). In addition, the economic heavy metal extraction was similar to the energy consumption, and its values decreased with increasing voltage strength since the energy consumption varied greatly under different electric field application treatments (0.03 to 2.90 kWh). Electric field application frequency and average daily application time, and increased with increasing electrode spacing, but the influence of all four electrochemical factors on EHME was insignificant (**Supplementary Table S5**).

**Figure 6:**
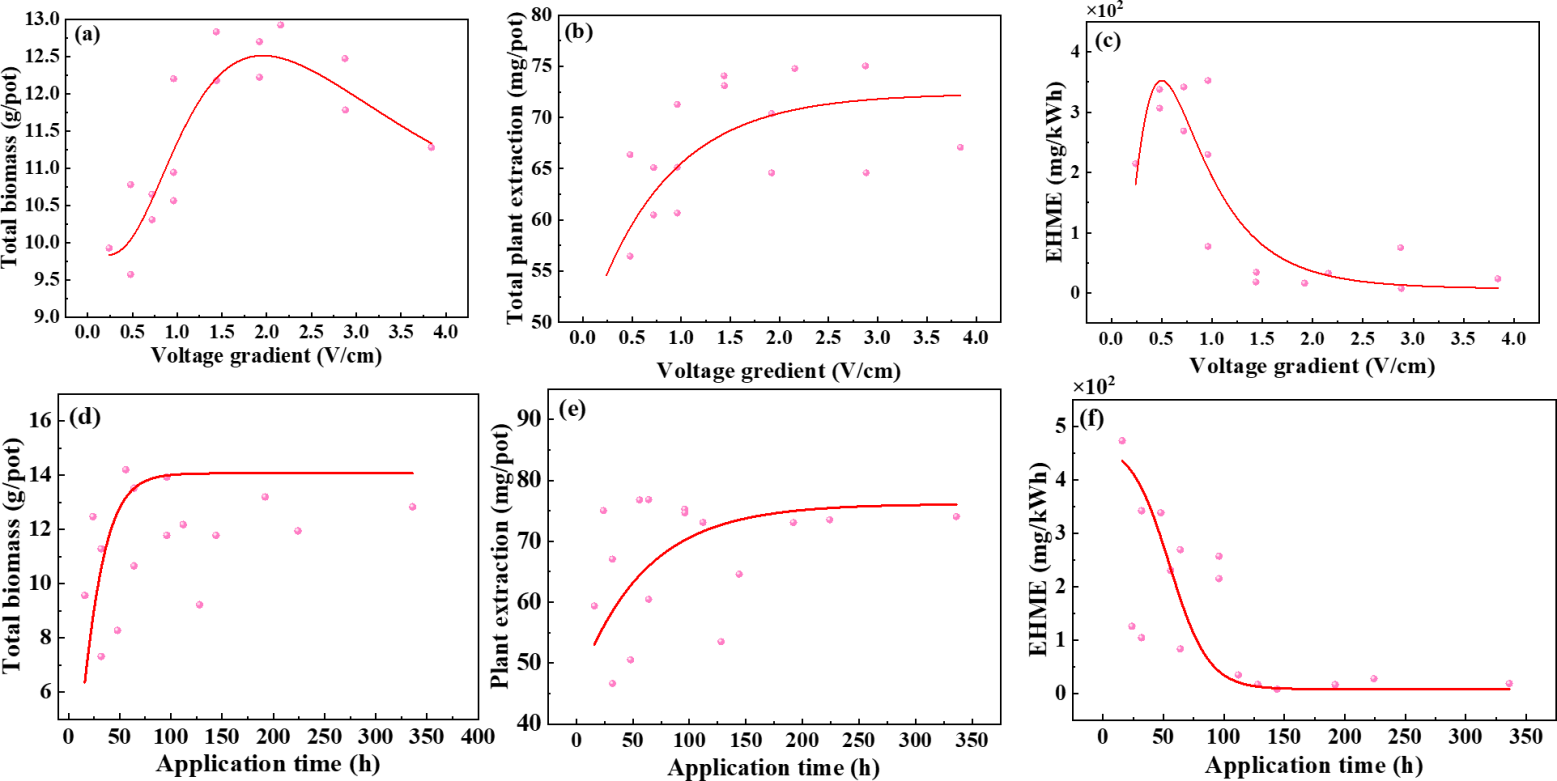
Effects difference of different voltage gradient and application time on biomass **(a, d)**, plant extraction **(b, e)** and EHME **(c, f)**.

There are differences in the differences of indicators by different factors, and a single factor cannot assess the effect of electric field parameters on the uptake of heavy metals by *S. plumbizincicola*. Therefore, the voltage and electrode spacing, application frequency and daily average application time were coupled into voltage gradient and application time to study its effect on biomass and heavy metal extraction. The biomass did not consistently increase with the voltage gradient and electric field application time, reaching a maximum at 2.5 V/cm and 100 h (**Figures 6a, d**). This is mainly due to excessive electric field can have obvious adverse effect on plant growth due to various electrochemical oxidation reactions and derivative effects (Liu et al., 2020), and a certain time of electric field application can promote plant growth, but too long time of electric field action can be harmful to plant growth and cause energy loss (Ma et al., 2024). Consequently, the best biomass promotion of *S. plumbizincicola* by electric field assistance was achieved when the voltage gradient was 2.5 V/cm and the electric field application time was 100h. Based on the high biomass principle, the better electro-assisted treatments were T10 and T11 treatments. As the voltage gradient increases, the heavy metal extraction rises rapidly, and it increases slowly and tends to stabilize when the voltage gradient exceeds 2.5 V/cm (**Figure 6b**). The reason for this can be revealed as the increase in voltage intensity increases the evaporation of soil moisture, leading to a more rapid decrease in moisture content and inhibiting the migration and dissociation of heavy metal ions (Zhou et al., 2020). Similarly, the heavy metal extraction increased with the electric field application time, but the extraction capacity tends to level off when the application time exceeds 100h (**Figure 6e**). Yan et al. (2019) compared the effects of remediation time, voltage gradient, electrode particle size and ratio on the removal of Cr^6+^ from soil by orthogonal experiments and found that remediation time had the greatest effect on Cr^6+^ removal by electrokinetic remediation. But excessive treatment time will not only not improve the removal rate of pollutants, but also cause useless power consumption (Han et al., 2014). For instance, the Pb accumulation of Indian mustard increased by 2–4 times and 2.81 times when treated with 2 V/cm DC electric field for 9d (30 or 60 min/d) or 16 d (8h/d), respectively (Lim et al., 2003; Cang et al., 2011). Generally, the total extraction of the four heavy metals by the *S. plumbizincicola* was the largest when the voltage gradient was 2.0 V/cm and electric field application time is 100 h. Based on the high heavy metal extraction principle, its better electro-assisted treatment is T3, T4 and T10 treatment. The EHME showed an increasing trend in the voltage gradient of 0.25-1.0 V/cm, slowly decreasing between 1.0-2.0 V/cm and then remaining constant (**Figure 6c**). The EHME decreases with the increase of electric field application time, and tends to be flat when electric field application time is 100h (**Figure 6f**). Romina et al. (2021) showed that when the soil voltage exceeds 50 V/m, there may be an increase in energy loss due to soil heating affecting electrical permeability, which in turn limits the effectiveness of the electrokinetic process. This study also confirmed that the removal of heavy metals did not significantly increase with the increase in remediation time, in agreement with the findings of Adhami et al. (2021). It can be seen that for different pollutants and soil conditions, reasonable control of the remediation time can ensure the effectiveness of remediation while also saving time and reducing costs. Based on the principle of high heavy metal extraction and low energy consumption, the better electro-assisted treatment is T3, T7, T8 and T12 treatments.

Overall, voltage gradient is considered to be the key indicators affecting the removal efficiency of EKAPR system due to the ability to influence enzyme activities, photosynthesis, plant growth metabolism and soil physicochemical properties. Appropriate voltage promotes plant biomass increase and root morphology development, and then improves heavy metal absorption capacity (Ma et al., 2024). However, the high intensity electric field will destroy the permeability and integrity of cell wall and damage the normal growth of plants (Janositz and Knorr, 2010), and the other side will limit the longitudinal movement of ions by reducing the trachea diameter, thereby inhibiting the absorption capacity of plants (Okumura et al., 2014; Lim et al., 2012). In addition, the electric field treatment time also significantly affected plant growth and heavy metal extraction. Prolonged application of electric field destroys the ultrastructure of root cells, resulting in decreased root biomass and root vigor (Xu et al., 2020; Sánchez et al., 2018). Previous studies have also shown that the single desorption of Pb decreases as time increases (Ma et al., 2022). Therefore, it is necessary to rationally regulate the electrokinetic parameters to balance the effects of electric field on the activation and directional migration of heavy metal and plant growth inhibition, so as to maximize their advantages. The most suitable voltage gradient and electric field application time in this study were 2.0 V/cm and 100h, respectively.4.3 Energy savings in EKAPR systems can be achieved by optimizing plant growth stages and electric field parameters.

In recent years, electrokinetic remediation has begun to develop on multiple aspects, and an increasing number of researchers have been working to improve pollutant removal efficiency by optimizing electric field configuration, introducing electrolytes, and combining with other remediation techniques. But in practical applications, factors such as focusing effects, electrode polarization, soil acidification/alkalization and continuous electric field application lead to high energy consumption (Zheng et al., 2024). Currently, researchers have mainly solved the problem of energy consumption in electric remediation systems by replacing the DC electric field with a pulsed electric field (Yuan et al., 2017), adjusting the position of the electrodes (Sun et al., 2019) and multi-electrode arrangement (Kim et al., 2021) to regulate the electric field, and developing the self-powered technology of solar cells (Jeon et al., 2015) and microbial fuel cells (Habibul et al., 2016). An example of this is Hassan et al. (2015) who found that electric remediation systems powered by solar cells can save up to 40% compared to grid power. Additionally, the researchers improved electric field parameters such as voltage gradient and current magnitude by using reference auxiliary electrodes to promote the migration of metal ions, thus reducing the output of electrical energy (Wang et al., 2019).

**Figure 7:**
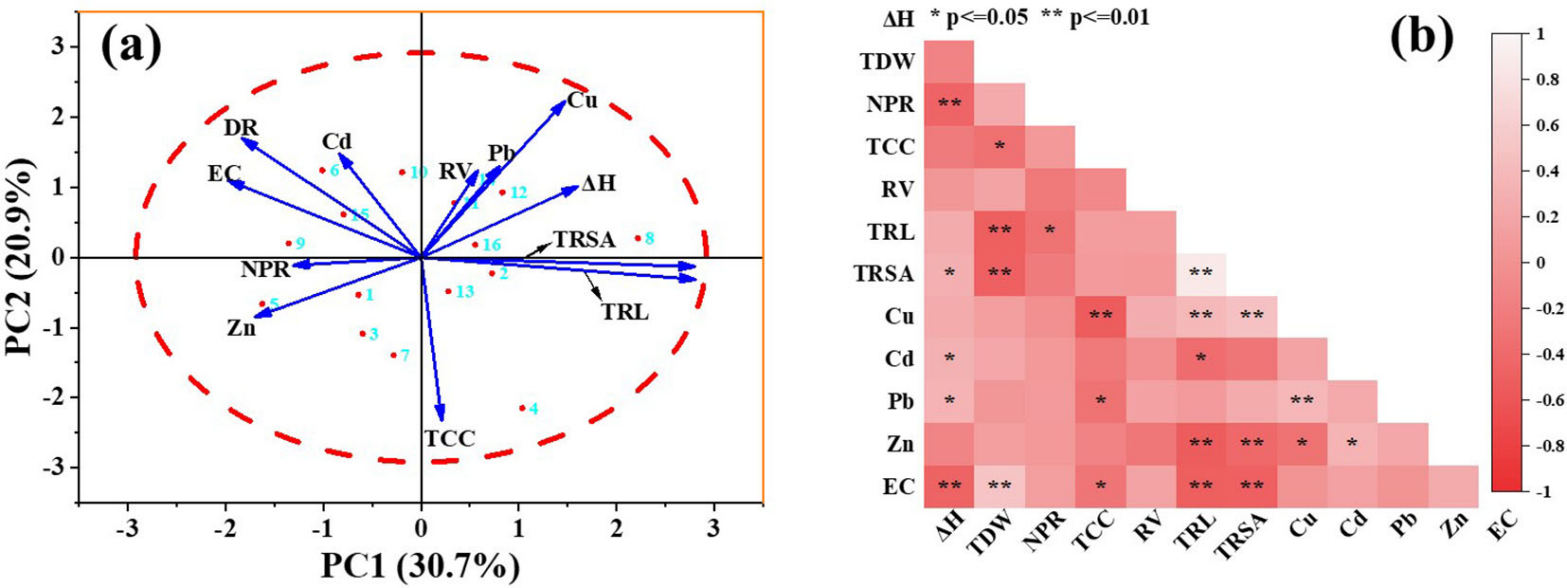
PCA biplots **(a)** and correlation plots (A) for the content of heavy metals influenced by plant growth index. (ΔH, plant height; TRL, Total Root Length; TRSA, Total Surface Area; RV, Root Vigor; DR, Dry weight; TCC, Total chlorophyll content; Cu, Total Cu extraction in plant; Zn, Total Zn extraction in plant; Cd, Total Cd extraction in plant; Pb, Total Pb extraction in plant).

In our study, the EKAPR system was optimized by both selecting the growth stages of the plants and adjusting the electric field parameters to achieve energy savings. Firstly, by comparing the growth and the heavy metals extraction of *S. plumbizincicola* with the electric field application at different growth periods, it was confirmed that the electric field application at a single growth period could effectively remove the heavy metals from the soil under the prerequisite of ensuring normal growth. According to the plant growth and heavy metal uptake and accumulation, the electric field treatment in the booming stage is beneficial to save the electric field treatment time while obtaining better remediation efficiency of the EKAPR system. Secondly, the correlation showed that energy consumption had a significant negative correlation with plant growth (ΔH, TCC, TRL, TRSA), and it no significant correlation with heavy metal content in plant (**Figure 7**). This result suggests that the remediation efficiency of the EKAPR system does not show a significant linear correlation with the energy consumption, rather there is an optimal value (Zhou et al., 2020), and it is necessary to optimize the electric field parameters to reduce the energy loss. As for example, the energy consumption increased from 0.03 kWh to 1.74 kWh by EKAPR system under T4-T6 treatment, but the total extraction of the four heavy metals decreased from 1.79 mg to 17.08 mg. The energy consumption of EKAPR system depends on the voltage strength, current density and electric field application time (**Supplementary Table S5**). Within a certain range, the electrode spacing changes the proton transfer distance in the solution, which in turn affects the system internal resistance and power output (Wang et al., 2017). The electric field frequency and the average daily application time together determine the electric field treatment time, and excessively long electric field times will significantly affect plant growth and heavy metal extraction (Fan et al., 2021). Therefore, we should reasonably balance the relationship between the heavy metals extraction by plants and the electrical energy power in order to obtain a high EKAPR system effect.

Overall, this study aimed to optimize the EKAPR system in terms of plant growth stages and electric field parameters for high heavy metal accumulation and low energy consumption, so as to expand the practical application value of the EKAPR system. This study is a novel and proven approach. Nonetheless, this recommended method cannot be used for all EKAPR systems due to differences of plant tolerance to electric field and growth stage. Therefore, comparative studies of different plants species should be extended in future studies to obtain a more comprehensive and accurate method of electric field application. Furthermore, this study only investigated the macro-level changes in plant growth and heavy metal accumulation under different treatments, and the physiological, microbiological, and molecular levels of plants should be further revealed.

## 5 Conclusion

To extend the practical application and reduce the energy consumption of the EKAPR system, this study optimized the electric field application strategy of the EKAPR system in terms of both plant growth stages and electric field parameters. The growth stages experiments showed that the heavy metal accumulation ability with electric field treatments under single growth stages were not less than those of whole growth period treatments, and it is best under the EK-B treatment. This is confirmed by the comprehensive scores for each treatment (0.70 for EK-B and only -0.03 for EK-W). These results suggested that the feasibility of short-term electric-assistance that can ensure the normal plant growth while effectively accumulating heavy metals. And electric field assistance at booming periods is an alternative method to save energy for replaces long-term electric field application. The electric field parameter optimization experiments show that four factors significantly (*p* ≤ 0.05) impacted the plant height and root morphology, but the heavy metals content in plants was not showing significant correlation with energy consumption. The biomass and heavy metal extraction of *S. plumbizincicola* were both better promoted at voltage gradient of 2.0 V/cm and total application time reached 100h. Also, the electric field application schemes are chosen differently according to different purposes. Based on the principles of high heavy metals accumulation increment and low energy consumption, the recommended electric field application strategy for *S. plumbizincicola* in the EKAPR system is to apply voltage intensity of 24 V, electrode spacing of 25 cm, electric field application frequency of 3 d intervals and average daily application time of 2 h in the booming period, and its EHME reaches 472.60 mg/kWh. To sum up, it is an alternative idea to save energy by optimizing both plant growth stages and electric field parameters to practice the principle of low carbon and broaden the application value of the EKAPR system. However, only the laboratory-scale application of *S. plumbizincicola* was demonstrated in this study, electric field parameter causing plant physiological differences, mechanism characterization or electrokinetic transport of contaminants needs further study.

## Data Availability

The original contributions presented in the study are included in the article/**Supplementary Material**. Further inquiries can be directed to the corresponding authors.
