# Supplementary material for: Regulated the electrokinetic application of different plant growth stages and parameters enhance the economic extraction of soil heavy metals
Source: Front Plant Sci. 2025 May 19;16:1557261. doi: 10.3389/fpls.2025.1557261 (PMC12127414; doi:10.3389/fpls.2025.1557261)
Supplement: Supplementary file 1 [file DataSheet1.docx]

**Supplementary materials**

**Table S1 The** **F-ratio and its probability obtained from the ANOVA analyses for the effects of treatments and for the orthogonal comparisons**

|  | ΔH (cm) | | Total dry weight (g/pot) | | Net photosynthetic rate | | Total chlorophyll content (μg/g) | |
| --- | --- | --- | --- | --- | --- | --- | --- | --- |
|  | F | P | F | P | F | P | F | P |
| A | 11.0 | 0.040^*^ | 2.40 | 0.25^ns^ | 0.67 | 0.63^ns^ | 0.39 | 0.77^ns^ |
| B | 9.66 | 0.047^*^ | 7.12 | 0.07^ns^ | 1.30 | 0.42^ns^ | 0.33 | 0.81^ns^ |
| C | 4.20 | 0.135^ns^ | 1.71 | 0.34^ns^ | 0.57 | 0.67^ns^ | 0.94 | 0.52^ns^ |
| D | 26.9 | 0.011^*^ | 1.41 | 0.39^ns^ | 0.92 | 0.53^ns^ | 0.13 | 0.93^ns^ |
|  | **Root vigor (ug/(g.h))** | | **Total root length (cm)** | | **Total root surface area (cm^2^)** | | **Root volume (cm^3^)** | |
|  | F | P | F | P | F | P | F | P |
| A | 0.78 | 0.58^ns^ | 2.81 | 0.21^ns^ | 1.09 | 0.47^ns^ | 17.3 | 0.02^*^ |
| B | 0.33 | 0.81^ns^ | 26.6 | 0.01^**^ | 31.0 | 0.01** | 11.4 | 0.04^*^ |
| C | 0.15 | 0.92^ns^ | 8.36 | 0.06^ns^ | 7.03 | 0.07^ns^ | 0.40 | 0.77^ns^ |
| D | 0.72 | 0.92^ns^ | 17.0 | 0.02^*^ | 14.6 | 0.03^*^ | 1.26 | 0.43^ns^ |

**Table S2 The range analysis of plant growth traits**

| **Indexs** |  | **A** | **B** | **C** | **D** | **Indexs** |  | **A** | **B** | **C** | **D** |
| --- | --- | --- | --- | --- | --- | --- | --- | --- | --- | --- | --- |
| **ΔH (cm)** | K_1_ | 3.41 | 2.92 | 3.18 | 4.39 | **Total dry weight (g/pot)** | K_1_ | 10.89 | 11.62 | 12.04 | 11.39 |
|  | K_2_ | 2.80 | 4.27 | 4.21 | 4.33 |  | K_2_ | 10.97 | 12.24 | 11.22 | 10.85 |
|  | K_3_ | 4.65 | 3.17 | 3.36 | 4.1 |  | K_3_ | 11.82 | 11.55 | 11.08 | 11.49 |
|  | K_4_ | 3.82 | 4.32 | 3.92 | 1.86 |  | K_4_ | 11.85 | 10.12 | 11.2 | 11.81 |
|  | R | 1.85 | 1.4 | 1.03 | 2.53 |  | R | 0.96 | 2.12 | 0.96 | 0.96 |
|  | Optimal Level | 3 | 4/2 | 2 | 1/2 |  | Optimal Level | 4 | 2 | 1 | 4 |
|  | Influence degree of factors | | | D＞A＞B＞C | |  | Influence degree of factors | | | B＞A=C=D | |
| **Net photosynthetic rate** | K_1_ | 5.72 | 3.62 | 7.08 | 3.17 | **Total chlorophyll content (μg/g)** | K_1_ | 790.9 | 745.6 | 725.3 | 742.1 |
|  | K_2_ | 6.68 | 5.9 | 4.18 | 3.51 |  | K_2_ | 731.5 | 635.2 | 676.6 | 762.6 |
|  | K_3_ | 4.09 | 6.79 | 4.09 | 6.59 |  | K_3_ | 693.8 | 820.4 | 703.2 | 710.4 |
|  | K_4_ | 3.2 | 3.39 | 4.34 | 6.43 |  | K_4_ | 714.2 | 729.1 | 825.2 | 715.2 |
|  | R | 3.48 | 3.4 | 2.99 | 3.42 |  | R | 97.1 | 185.2 | 148.6 | 52.2 |
|  | Optimal Level | 2 | 3 | 1 | 3 |  | Optimal Level | 1 | 3 | 4 | 2 |
|  | Influence degree of factors | | | A≈D≈B＞C | |  | Influence degree of factors | | | B＞C＞A＞D | |
| **Root vigor (ug/(g.h))** | K_1_ | 233.8 | 391 | 415 | 458.9 | **Total root length (cm)** | K_1_ | 81.4 | 56.72 | 72.75 | 72.83 |
|  | K_2_ | 460.4 | 369.9 | 454.4 | 579.9 |  | K_2_ | 79.22 | 80.43 | 59.63 | 104.6 |
|  | K_3_ | 576.1 | 572.8 | 506.8 | 245.9 |  | K_3_ | 65.04 | 60.29 | 84.05 | 59.90 |
|  | K_4_ | 461.9 | 398.5 | 356.1 | 447.6 |  | K_4_ | 81.34 | 109.6 | 90.57 | 69.70 |
|  | R | 342.3 | 202.95 | 150.775 | 333.95 |  | R | 16.36 | 52.88 | 30.94 | 44.70 |
|  | Optimal Level | 3 | 3 | 3 | 2 |  | Optimal Level | 1 | 4 | 4 | 2 |
|  | Influence degree of factors | | | A≈D＞B＞C | |  | Influence degree of factors | | | B＞D＞C＞A | |
| **Total root surface area (cm^2^)** | K_1_ | 9 | 7.12 | 8.89 | 8.21 | **Root volume (cm^3^)** | K_1_ | 0.45 | 0.24 | 0.30 | 0.30 |
|  | K_2_ | 8.4 | 8.28 | 7.39 | 10.6 |  | K_2_ | 0.27 | 0.35 | 0.31 | 0.35 |
|  | K_3_ | 8.36 | 7.97 | 9.31 | 7.89 |  | K_3_ | 0.24 | 0.26 | 0.30 | 0.29 |
|  | K_4_ | 8.96 | 11.4 | 9.14 | 8.05 |  | K_4_ | 0.28 | 0.4 | 0.33 | 0.30 |
|  | R | 0.64 | 4.28 | 1.92 | 2.71 |  | R | 0.21 | 0.16 | 0.03 | 0.06 |
|  | Optimal Level | 1 | 4 | 3 | 2 |  | Optimal Level | 1 | 4 | 4 | 2 |
|  | Influence degree of factors | | | B＞D＞C＞A | |  | Influence degree of factors | | | A＞B＞D＞C | |

K_1_, K_2_, K_3_ and K_4_ were the average values of Level 1, Level 2, Level 3 and Level 4 for each factor, and the range (R) was the difference between the maximum and minimum average for each factor. And A, B,C and D represent electric field strength, electrode spacing, frequency of electric field applied and application time, respectively. The same below

**Table S3 The range analysis of average heavy metal content in plant (mg/kg)**

| **Indexs** |  | **A** | **B** | **C** | **D** | **Indexs** |  | **A** | **B** | **C** | **D** |
| --- | --- | --- | --- | --- | --- | --- | --- | --- | --- | --- | --- |
| **Cu** | K_1_ | 16.04 | 15.36 | 20.84 | 18.65 | **Cd** | K_1_ | 669.6 | 741.4 | 757.3 | 778.8 |
|  | K_2_ | 18.88 | 21.56 | 17.76 | 19.78 |  | K_2_ | 764.2 | 756.3 | 748.5 | 721.3 |
|  | K_3_ | 19.61 | 17.06 | 20.08 | 17.74 |  | K_3_ | 767.7 | 745.6 | 735.3 | 766.6 |
|  | K_4_ | 20.67 | 21.24 | 16.52 | 19.03 |  | K_4_ | 755.4 | 713.7 | 715.8 | 690.2 |
|  | R | 4.63 | 6.20 | 4.32 | 2.04 |  | R | 98.1 | 42.6 | 41.5 | 88.6 |
|  | Optimal Level | 4 | 2/4 | 1 | 2 |  | Optimal Level | 3/2 | 2 | 1 | 1 |
|  | Influence degree of factors | | | B＞A＞C＞D | |  | Influence degree of factors | | | A＞D＞B＞C | |
| **Pb** | K_1_ | 32.90 | 30.63 | 36.00 | 30.79 | **Zn** | K_1_ | 6245.5 | 6575.1 | 6148.3 | 6026.3 |
|  | K_2_ | 30.87 | 34.19 | 30.82 | 36.37 |  | K_2_ | 6139.8 | 5802.8 | 5867.7 | 5790.1 |
|  | K_3_ | 37.00 | 31.00 | 32.33 | 30.67 |  | K_3_ | 6044.9 | 5889.5 | 6081.5 | 6283.1 |
|  | K_4_ | 27.81 | 32.76 | 29.42 | 30.75 |  | K_4_ | 5636.2 | 5799.0 | 5968.8 | 5966.8 |
|  | R | 9.19 | 3.56 | 6.58 | 5.70 |  | R | 609.3 | 776.1 | 280.6 | 493.0 |
|  | Optimal Level | 3 | 2 | 1 | 2 |  | Optimal Level | 1 | 1 | 1 | 3 |
|  | Influence degree of factors | | | A＞C＞D＞B | |  | Influence degree of factors | | | B＞A＞D＞C | |

**Table S4 The range analysis of heavy metal extraction (mg)**

| **Indexs** |  | **A** | **B** | **C** | **D** | **Indexs** |  | **A** | **B** | **C** | **D** |  |
| --- | --- | --- | --- | --- | --- | --- | --- | --- | --- | --- | --- | --- |
| **Cu** | K_1_ | 0.118 | 0.129 | 0.152 | 0.138 | **Cd** | K_1_ | 8.021 | 7.777 | 9.816 | 9.933 |  |
|  | K_2_ | 0.123 | 0.140 | 0.151 | 0.161 |  | K_2_ | 10.13 | 10.22 | 10.13 | 10.67 |  |
|  | K_3_ | 0.170 | 0.153 | 0.130 | 0.127 |  | K_3_ | 10.59 | 11.90 | 9.094 | 9.288 |  |
|  | K_4_ | 0.159 | 0.148 | 0.136 | 0.143 |  | K_4_ | 11.41 | 10.26 | 11.12 | 10.27 |  |
|  | R | 0.052 | 0.024 | 0.022 | 0.034 |  | R | 3.389 | 4.123 | 2.026 | 1.382 |  |
|  | Optimal Level | 3 | 3 | 1/2 | 2 |  | Optimal Level | 4 | 3 | 4 | 2 |  |
|  | Influence degree of factors | | | A＞D＞B＞C | |  | Influence degree of factors | | | B＞A＞C＞D | | |
| **Pb** | K_1_ | 0.330 | 0.317 | 0.336 | 0.349 | **Zn** | K_1_ | 51.59 | 45.01 | 52.66 | 53.67 |  |
|  | K_2_ | 0.303 | 0.312 | 0.363 | 0.342 |  | K_2_ | 52.71 | 56.21 | 56.53 | 57.90 |  |
|  | K_3_ | 0.372 | 0.376 | 0.316 | 0.337 |  | K_3_ | 56.10 | 58.67 | 52.74 | 51.44 |  |
|  | K_4_ | 0.362 | 0.362 | 0.352 | 0.339 |  | K_4_ | 57.47 | 57.98 | 55.93 | 54.86 |  |
|  | R | 0.069 | 0.064 | 0.047 | 0.012 |  | R | 5.88 | 13.66 | 3.87 | 6.46 |  |
|  | Optimal Level | 3 | 3 | 2 | 1/2 |  | Optimal Level | 4 | 3 | 2 | 2 |  |
|  | Influence degree of factors | | | A＞B＞C＞D | |  | Influence degree of factors | | | B＞D＞A＞C | | |

**Table S5 Variance analysis and range analysis of energy consumption (kW.h)**

|  | **F** | **P** |  | **A** | **B** | **C** | **D** |
| --- | --- | --- | --- | --- | --- | --- | --- |
| A | 20.02 | .017* | K_1_ | 0.31 | 5.42 | 3.44 | 0.68 |
| B | 18.55 | .019* | K_2_ | 3.17 | 3.23 | 3.21 | 2.05 |
| C | 2.123 | .276^ns^ | K_3_ | 4.86 | 2.76 | 3.46 | 3.09 |
| D | 31.11 | .009** | K_4_ | 3.89 | 0.82 | 2.12 | 6.41 |
|  |  |  | R | 4.55 | 4.60 | 1.34 | 5.73 |
|  |  |  | Optimal Level | 3 | 1 | 3/1 | 4 |
|  |  |  | Influence degree of factors | | D＞B≈A＞C | |  |

**Table S6 Variance analysis and range analysis of EHME (mg/kW.h)**

|  | **F** | **P** |  | **A** | **B** | **C** | **D** |
| --- | --- | --- | --- | --- | --- | --- | --- |
| A | 4.501 | .124 ^ns^ | K_1_ | 255.1 | 69.61 | 77.55 | 279.9 |
| B | 2.832 | .208 ^ns^ | K_2_ | 211.4 | 98.90 | 160.9 | 166.3 |
| C | 1.154 | .455 ^ns^ | K_3_ | 104.3 | 207.5 | 181.9 | 95.91 |
| D | 4.120 | .138 ^ns^ | K_4_ | 35.73 | 230.4 | 186.1 | 64.29 |
|  |  |  | R | 219.4 | 160.8 | 108.6 | 215.6 |
|  |  |  | Optimal Level | 1 | 4 | 4 | 1 |
|  |  |  | Influence degree of factors | | A＞D＞B＞C | |  |

**
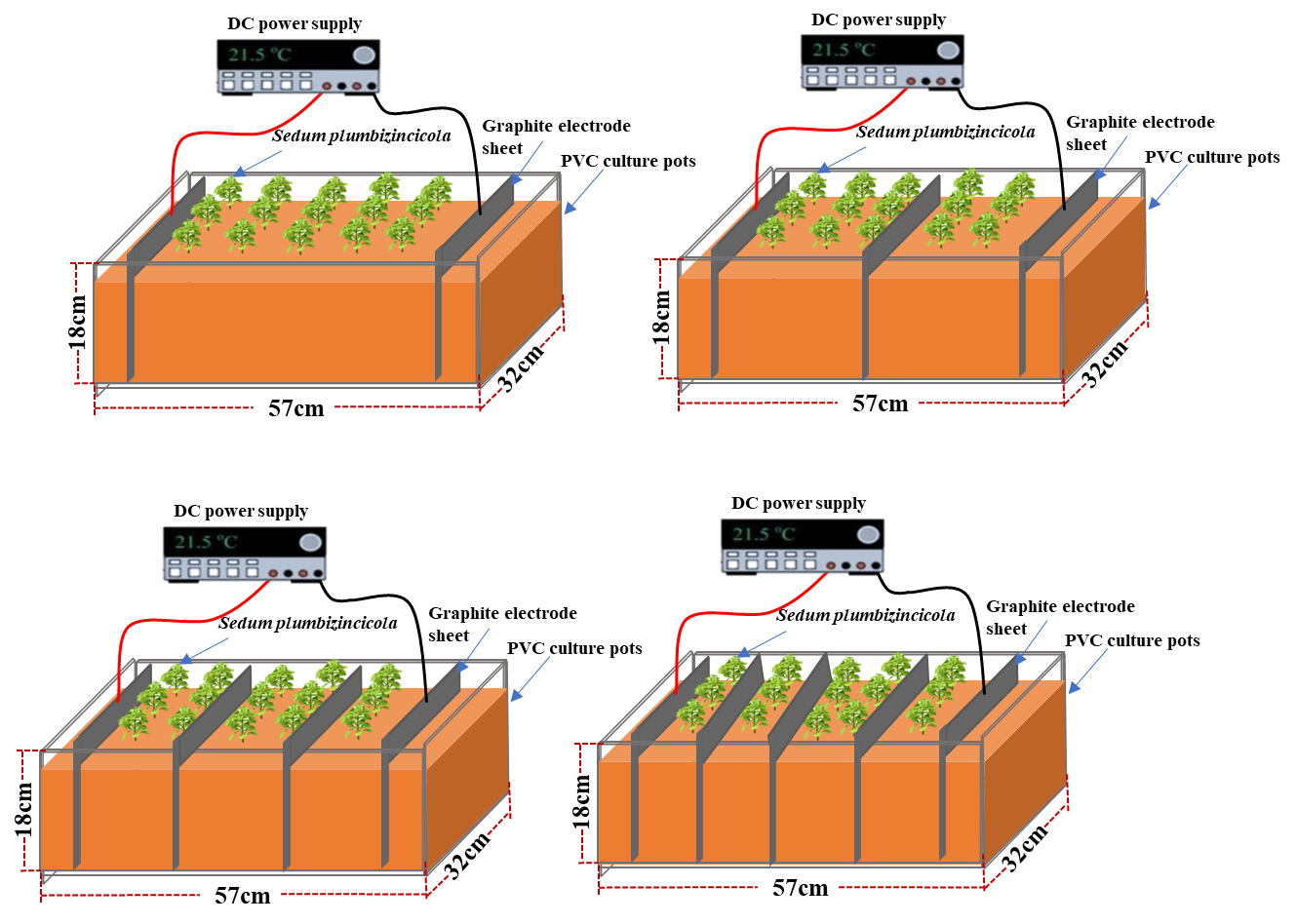
**

**Fig. S1 Potted setup diagram for orthogonal experiments**
